# Cultured Myoblasts Derived from Rat Soleus Muscle Show Altered Regulation of Proliferation and Myogenesis during the Course of Mechanical Unloading

**DOI:** 10.3390/ijms23169150

**Published:** 2022-08-15

**Authors:** Margarita Y. Komarova, Sergey V. Rozhkov, Oksana A. Ivanova, Olga V. Turtikova, Timur M. Mirzoev, Renata I. Dmitrieva, Boris S. Shenkman, Natalia A. Vilchinskaya

**Affiliations:** 1 Myology Laboratory, Institute of Biomedical Problems RAS, 123007 Moscow, Russia; 2 Institute of Molecular Biology and Genetics, Almazov National Medical Research Centre, 197341 Saint Petersburg, Russia

**Keywords:** soleus muscle, mechanical unloading, myoblast, MRF, proliferation, differentiation, myogenesis, transcriptome analysis

## Abstract

The structure and function of soleus muscle fibers undergo substantial remodeling under real or simulated microgravity conditions. However, unloading-induced changes in the functional activity of skeletal muscle primary myoblasts remain poorly studied. The purpose of our study was to investigate how short-term and long-term mechanical unloading would affect cultured myoblasts derived from rat soleus muscle. Mechanical unloading was simulated by rat hindlimb suspension model (HS). Myoblasts were purified from rat soleus at basal conditions and after 1, 3, 7, and 14 days of HS. Myoblasts were expanded in vitro, and the myogenic nature was confirmed by their ability to differentiate as well as by immunostaining/mRNA expression of myogenic markers. The proliferation activity at different time points after HS was analyzed, and transcriptome analysis was performed. We have shown that soleus-derived myoblasts differently respond to an early and later stage of HS. At the early stage of HS, the proliferative activity of myoblasts was slightly decreased, and processes related to myogenesis activation were downregulated. At the later stage of HS, we observed a decrease in myoblast proliferative potential and spontaneous upregulation of the pro-myogenic program.

## 1. Introduction

Skeletal muscle is a highly plastic tissue that has a unique ability to regenerate following injury. Skeletal muscle regeneration is possible due to a special type of small mononuclear cells called satellite cells (SCs), which are located at the periphery of adult skeletal muscle fibers (between the plasma membrane of a muscle fiber and the surrounding basal lamina) [1]. In undamaged muscle under normal conditions, SCs are quiescent, they neither proliferate nor differentiate. In response to injury/mechanical strain, SCs become activated; they proliferate, differentiate and then fuse to damaged myofibers or to each other to form new myofibers [2]. At the same time, following activation, proliferation and early stage of differentiation, a small part of SCs exits the cell cycle and becomes quiescent; this mechanism maintains the stability of the pool of SCs that supports skeletal muscle regenerative potential over the lifetime.

Under conditions of skeletal muscle degeneration (muscle dystrophy/atrophy), there is a decline in muscle regenerative capacity due to a decrease in functional properties of muscle SCs [2,3,4]. For example, in Duchenne muscular dystrophy (DMD), muscle fiber degenerative processes are associated with continuous SCs activation not resulting in complete muscle recovery, which was estimated based on the heterogeneity in fiber diameter and an increased percentage of fibers with centralized nuclei [5,6,7]. Also, a spontaneous reactivation of developmental programs in adult skeletal muscle was detected in regenerating muscles after injury or under pathological conditions such as muscular dystrophy, different types of myopathies or muscle wasting induced by metabolic disorders [8,9]. Therefore, the pathological alterations in the functional properties of SCs in atrophying muscle are relatively well described for skeletal muscle myopathies of different genesis [2,9,10,11,12], but not for muscle atrophy caused by mechanical unloading/simulated microgravity.

Under real or simulated microgravity, the structure and function of postural muscle fibers (such as soleus muscle) undergo substantial remodeling. Postural muscle dysfunction occurs during the first hours of gravitational unloading [13,14], leading to changes in signaling pathways that control protein synthesis and breakdown, expression of contractile and regulatory proteins, and energy metabolism [15,16].

To date, there is only scarce data on the effects of gravitational unloading on the functional activity of muscle SCs. It has been demonstrated that gravitational unloading for 7 and 14 days leads to a decrease in the total number of SCs in rat soleus muscle [17,18,19] and murine soleus, gastrocnemius and plantaris muscles [20].

Also, it has been shown that 14-day hindlimb unloading can significantly attenuate proliferation and differentiation of SCs in murine soleus muscle following injury [18,21]. On the other hand, an exposure of mice to 30-day microgravity conditions (BION-M1 spaceflight) does not appear to affect regenerative capacity and SC differentiation in quadriceps muscle [22]. Moreover, 7-day hindlimb unloading results in the activation of SC proliferation (as assessed by bromodeoxyuridine (BrdU) labeling) in mouse gastrocnemius muscle [23]. It has also been demonstrated that hindlimb immobilization for 7 days results in the activation of muscle SCs with a concomitant decrease in total number of SCs [24].

Although a number of reports have described an impact of real or simulated microgravity on the number of muscle SC, unloading-induced changes in the functional activity of skeletal muscle SCs remain poorly studied. The purpose of our study was to investigate how both short-term (1–3 days) and long-term (7–14 days) mechanical unloading would affect cultured myoblasts derived from rat soleus muscle. To this end, proliferative activity and expression of genes regulating differentiation and fusion were analyzed in soleus-derived myoblasts expanded in vitro. It is important to note that our study was exclusively focused on the initial stage of development of the soleus-derived myoblasts (24 h after purification/pre-plating).

## 2. Results

### 2.1. Soleus Muscle Weight and Myogenic Differentiation of Myoblasts

**Figure 1:**
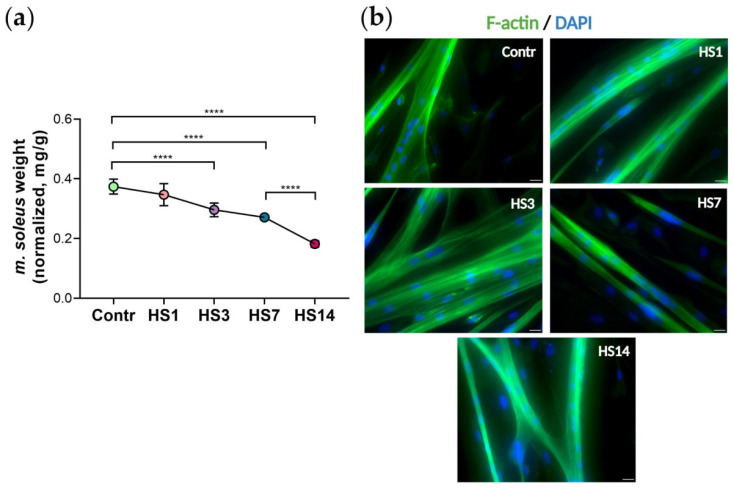
Changes in soleus muscle weight following 1, 3, 7 and 14 days of mechanical unloading (HS): (**a**) rat soleus muscle weight normalized to the body weight; *n* = 16, **** *p* < 0.0001; (**b**) Myogenic differentiation (the 5th day of differentiation) of myoblasts purified from control muscle and hindlimb suspended muscle (HS1, HS3, HS7, HS14). Myotube formation was visualized with F-actin antibody; scale bar = 20 µm.

In this study, using a hindlimb suspension (HS) model [25], we have analyzed how mechanical unloading influences functional properties of soleus muscle-derived cultured myoblasts. The hindlimb suspension lasted for 1, 3, 7, and 14 days. A significant decrease in rat soleus weight after 3, 7, and 14 days of HS confirmed the development of muscle atrophy (Figure 1a).

Primary myoblasts were derived from rat soleus muscle as described in previous works [9], then expanded in vitro for 24 h after purification (pre-plating). All measurements presented in the current study were performed at this early time-point of myoblast cultivation, i.e., 24 h following purification. However, in order to confirm the myogenic nature of the cultured cells, stimulation of cell differentiation was performed (Figure 1b). On the 5th day of differentiation, we observed that myoblasts derived from soleus muscle following unloading formed myotubes with normal striated structure. This clearly confirmed that during myoblast isolation and in vitro expansion the myogenic nature of the cells was well-preserved (representative microphotographs are shown in Figure 1b).

### 2.2. The Proliferation Activity and Myogenic Commitment of Myoblasts Isolated from Rat Soleus after Mechanical Unloading

**Figure 2:**
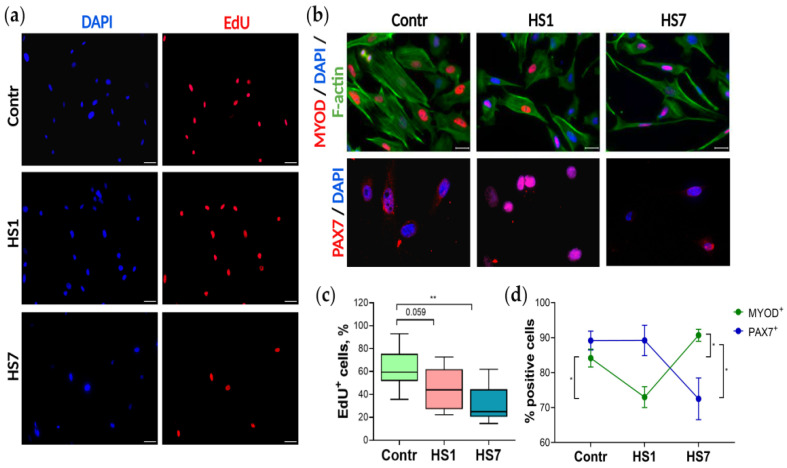
Functional properties of myoblasts cultured for 24 h after purification: (**a**) Myoblast proliferative activity analysis by EdU incorporation; EdU^+^ cells are shown in red; nuclei are shown in blue. (**b**) Immunostaining: MYOD and PAX7 positive cells are shown in red; nuclei are shown in blue; F-actin is shown in green; scale bar = 20 µm. (**c**) The percentage of EdU^+^ cells estimated in 2 independent experiments; *n* > 200, ** *p* < 0.01. (**d**) The percentage of MYOD positive cells increases in the HS7 cultures, in contrast to PAX7 positive cells; *n* > 200, * *p* < 0.05.

In order to determine myoblast proliferation activity, cells derived from the Control, HS1 and HS7 muscles were cultured with EdU, and the percentage of EdU-positive proliferating cells was estimated after overnight treatment (Figure 2a,c). We have shown that EdU incorporation was substantially lower in cultures derived from the HS7 muscle than in cultures derived from the Control and HS1 muscles, indicating the decreased proliferation activity: while in the control cultures almost 60% of myoblasts were in proliferative phase, in the HS1 cultures proliferating fraction decreased to 50%, and in the HS7 cultures only 20% of EdU^+^ proliferating cells were detected (Figure 2c). In short, myoblasts isolated from atrophying muscle showed substantially decreased proliferation rate, and this effect correlated with the duration of mechanical unloading.

As a rule, stem cell proliferation rate decreases as cells enter a differentiation stage, and altered coordination in the regulation of proliferation/differentiation balance can serve as one of the markers of skeletal muscle wasting [10,12]. Therefore, we have tested if spontaneous myogenic commitment was activated in the HS1 and HS7 myoblast cultures.

**Figure 3:**
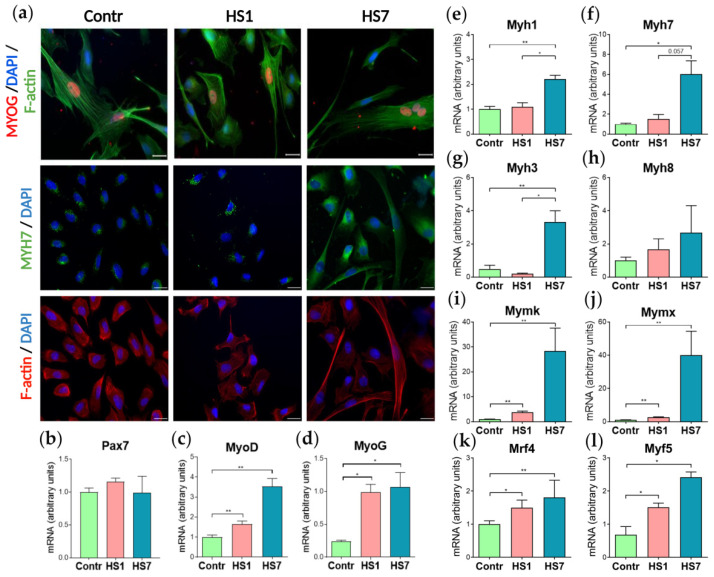
The effect of skeletal muscle functional unloading on myogenic factors expression in myoblasts cultured for 24 h after purification: (**a**) Immunostaining for slow myosin MYH7 in cultured myoblasts: MYH7—green, F-actin red; MYOG in non-differentiated myoblasts: MYOG red; F-actin green; nuclei blue; scale bar = 20 µm; F-actin staining was used as a positive control; (**b**–**l**) mRNA expression analysis: *Pax7* (a marker of satellite cells); *MyoD* and *MyoG* (myogenic regulatory factors); *Myh7* (slow-twitch myosin); *Myh3* (embryonic myosin); *Myh1* (fast-twitch myosin); *Myh8* (fetal skeletal muscle myosin); *Mymk* and *Mymx* (myoblasts fusion markers); *Mrf4* (myogenic regulatory factor); *Myf5* (myogenic regulatory factor); (*n* = 4; * *p* < 0.05; ** *p* < 0.01).

PAX7 is a marker of SCs in skeletal muscle, and we have detected *Pax7* expression in all myoblast cultures (Figure 3b and Figure S1), but the fraction of PAX7^+^ cells differed between the cultures: in the control and HS1 cultures we detected about 90% of PAX7^+^ cells, while in the HS7 cultures only 70% of myoblasts expressed PAX7 (Figure 2b,d). At the same time, using immunostaining, in all the cultures we detected the expression of myogenic marker MYOD, the expression of which increases when myoblasts are committed to differentiate [26] (Figure 2b,d). The calculation of MYOD^+^ fraction in all the cultures revealed the dynamics opposite to one for PAX7^+^ cells: in the HS1 cultures we detected a significant decrease in MYOD^+^ cells, while in the HS7 cultures this fraction significantly increased compared to the control values (Figure 2d). To summarize, the analysis of PAX7^+^ and MYOD^+^ cellular subpopulations showed that the PAX7^+^/MYOD^+^ composition differs between the Control/HS1/HS7 cultures. While in the HS1 samples the fraction of PAX7^+^ cells were substantially greater than the fraction of MYOD^+^ cells; in the HS7 samples the fraction of PAX7^+^ cells dropped down significantly and the fraction of MYOD^+^ cells increased up to 90%, indicative of an increase in subpopulation of myoblasts committed to differentiate between day 1 and day *7* of HS (Figure 2d). The immunostaining for another myogenic transcription factor, MYOG, that controls early steps of differentiation including myoblasts fusion [26], also confirmed its expression in all the cultures. Both immunostaining and qRT-PCR analysis showed an increased expression of slow-type myosin in the HS7 cultures but not in the control and HS1 samples (Figure 3a).

RT-PCR analysis revealed that mRNA expression levels of *Pax7* did not differ between the studied cultures (Figure 3b), while mRNA expression levels of both *MyoD* and *MyoG* significantly increased in the HS1 cultures (1.6-fold and 4-fold, respectively) and the HS7 cultures (3.5-fold and 4.5-fold, respectively) compared to the control cultures (Figure 3c,d). In the 7HS cultures, mRNA expression levels of *Myh1*, *Myh7* and *Myh3* were significantly upregulated by 2.2-fold, 6-fold and 6.9-fold, respectively (Figure 3e–g). Expression levels of the fusion markers, *Mymk* and *Mymx*, slightly increased in the HS1 cultures (3.7-fold and 2.6-fold, respectively) and greatly increased in the HS7 cultures (27.6-fold and 40-fold, respectively) compared to the control myoblasts (Figure 3i,j). *Mrf4* and *Myf5* expression levels were upregulated in the HS1 cultures (1.5-fold and 2.2-fold, respectively) as well as in the HS7 cultures (1.8-fold and 3.5-fold, respectively) compared to the control cultures (Figure 3k,l). Together, these data show that myoblasts (cultured for 24 h after purification) derived from unloaded/disused soleus muscles demonstrate spontaneous activation of differentiation and fusion markers.

### 2.3. Transcriptome Analysis Reveals Two Phases in Soleus-Derived Myoblasts’ Response to Mechanical Unloading

In order to reveal more details regarding myoblasts’ response to skeletal muscle mechanical unloading, we added two more time-points for consideration, day 3 (HS3) and day 14 (HS14). The HS3 time point was added in order to test how rapidly a strong level of activation of the myogenic commitment was achieved after the onset of HS. The HS14 point was added to find out if myoblast activation was transitory or remained stable for a longer period of time.

**Figure 4:**
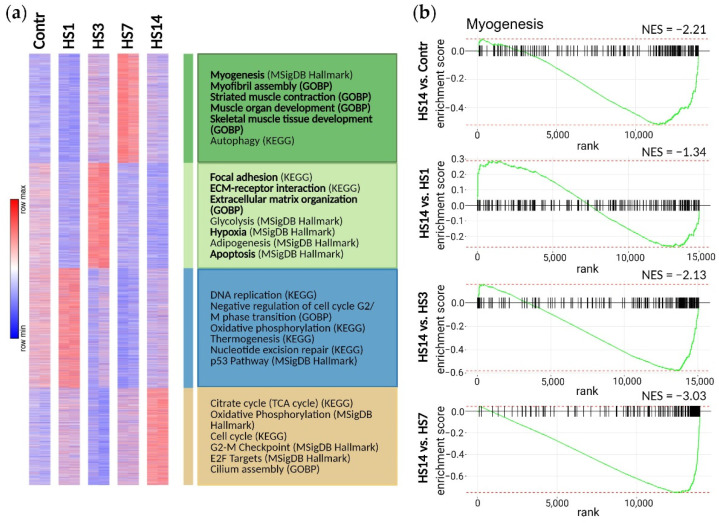
A transcriptome analysis revealed a change in myogenic potential at different time-points of mechanical unloading: (**a**) K-means clustering analysis of 15,829 genes in the Contr, HS1, HS3, HS7 and HS14 samples; (**b**) GSEA enrichment plots of the hallmark myogenesis pathway over ranked genes between possible pairs of cultured myoblasts: Contr, HS1, HS3, HS7 and HS14; *p* < 0.01, normalized enrichment scores (NES) are shown.

To elucidate molecular pathways behind differences between myoblasts isolated from unloaded soleus muscles at different time points, we performed transcriptome sequencing, and co-regulated genes were analyzed using k-means clustering. Four major clusters were identified, based on patterns of expression at different time-points after HS. Each cluster was characterized by upregulation in the specific sample (Figure 4a). Within these clusters, pathways associated with myogenesis were identified in cluster 1: genes in this cluster were upregulated in the HS7 samples.

Gene Set Enrichment Analysis (GSEA) enrichment plots of the hallmark myogenesis pathways over ranked genes between the control cells and the HS1/HS3/HS7/HS14 cultures are shown in Figure 4b. Interestingly, in spite of increased expression of the specific myogenic factors, such as *MyoG*, *MyoD*, *Mymk* and *Mymx*, in the HS1 cultures (Figure 3), the GSEA enrichment plot shows a significant downregulation of global myogenesis pathway in the HS1 cultures (Figure 4b), which is in a good agreement with a decrease in MYOD^+^ cellular subpopulation in the HS1 cultures (Figure 2d). A significant upregulation of global myogenesis pathways was shown in the HS3 cells with further upregulation in the HS7 cultures (Figure 4b). However, in the HS14 cultures the GSEA enrichment plot showed a significant downregulation of myogenesis. Together, these data show that the HS3 myoblast cultures were clearly activated for myogenic differentiation, and this activation increased further in the HS7 cultures. It seems, however, that this activation is not a continuous response to unloading, but just an acute reaction to the external signal that disappears by the 14th day of HS. The results of qPCR analysis of the genes that regulate different steps of myogenesis are given in Supplementary Figure S1.

**Figure 5:**
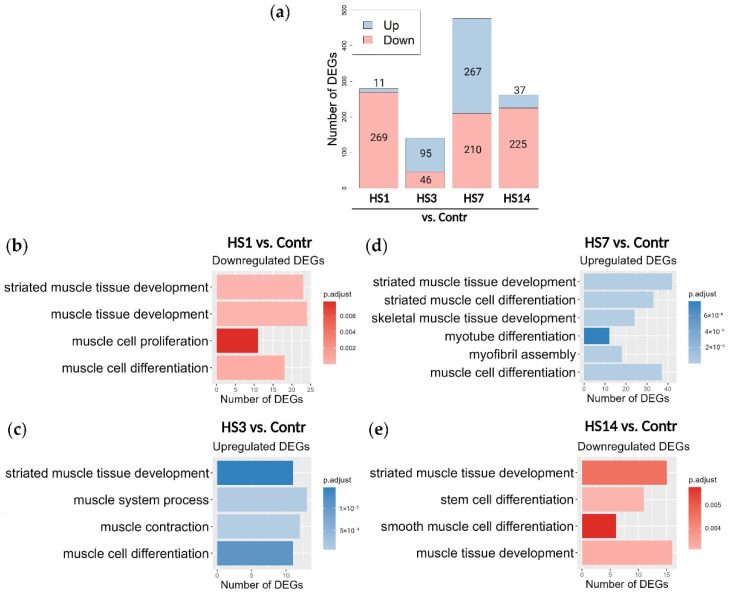
Differentially expressed genes associated with muscle regeneration and differentiation processes: (**a**) Number of up- and downregulated differentially expressed genes (DEGs) found in all four pairs (HS vs. Control); log2 fold change > 1.5, *p* < 0.01; (**b**–**e**) GO Enrichment Analysis of upregulated and downregulated DEGs using GO Biological Process database.

We further determined the differentially expressed genes (DEGs) between the various HS myoblast cultures and the control samples: HS1 vs. Control, HS3 vs. Control, HS7 vs. Control and HS14 vs. Control (Figure 5a). We found that in the HS1 and HS14 cultures most of the DEGs were downregulated, while in the HS3 and HS7 cultures the substantial fractions of both up- and downregulated DEGs were detected.

To identify the biological functions associated with the DEGs at different time points GO Enrichment Analysis was performed. We observed that the effect of 1- and 7-day HS on soleus-derived cultured myoblasts substantially differed, while the HS3 cells showed more similarities with the HS7 cells but not with the HS1 cells. All pathways associated with the DEGs are summarized in Supplementary Table S1. In addition, selected results related to signaling pathways involved in the regulation of muscle regeneration and myoblast differentiation are shown in Table 1, Table 2 and Table 3 and in Figure 5b,c.

As expected, and based on the results shown in Figure 4, a substantial proportion of upregulated genes in the HS3 and HS7 samples was found to belong to the pathways that regulate different brunches of skeletal muscle differentiation and function, while in the HS1 and HS14 samples pathways regulating muscle differentiation were downregulated (Figure 5a–e). Importantly, we can see here that in the HS7 cultures more upregulated DEGs belong to the pathways that regulate myogenesis compared to the HS3 cultures, and pathways upregulated in the HS7 cultures are associated with more advanced steps of myogenesis such as myofibril assembly and myotube differentiation (Figure 5c,d), indicating a strong level of activation of myoblast commitment. The downregulation of muscle cell proliferation pathway in the HS1 culture (Figure 5b) was also in a good agreement with the results of our functional experiments that showed both a decrease in myoblast proliferative activity and a decrease in MYOD^+^ subpopulation (Figure 2c,d).

**Figure 6:**
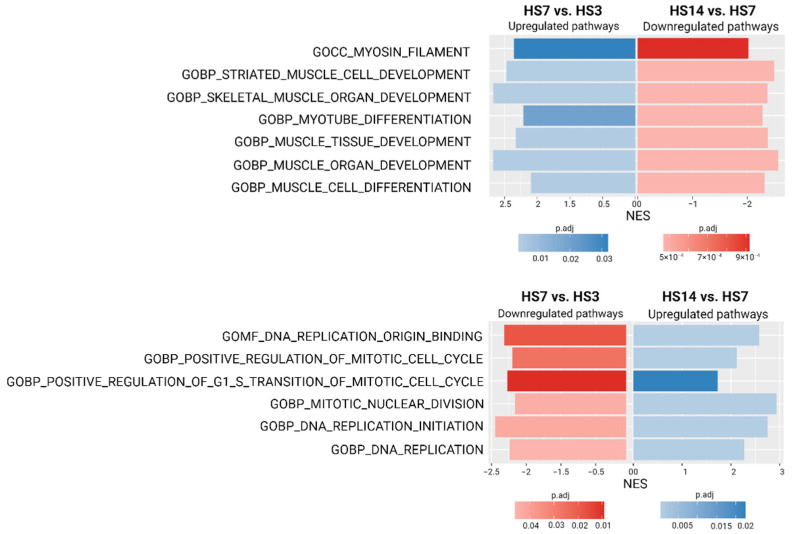
The fast gene set enrichment analysis (FGSEA) of all identified genes using the GO Biological Process database. Results visualized as bar plot of normalized enrichment score (NES) that display significant up/downregulated pathways that regulate the progress of cells through the cell cycle and the activation of the myogenic program in the early and late stages of unloading.

Transcriptome analysis also confirmed the diverse effects of the early and later stages of mechanical unloading on the soleus-derived cultured myoblasts. We have shown the reciprocal changes in pathways that regulate the progression of cells through cell cycle (proliferation activity) and pathways that regulate the activation of myogenic program in myoblasts derived from unloaded muscle (Figure 6). While during the transition from day 3 to day 7 of unloading upregulation of the pro-myogenic pathways and downregulation of the pathways responsible for proliferative activity was detected, during the transition from day 7 to day 14 of unloading the opposite tendency was observed, indicating substantial changes in the cell status in the course of mechanical unloading (Figure 6).

The full list of the DEGs, which were significantly different between samples is given in Supplementary Table S1; principal component analysis (PCA) is shown in Figure S2; gene expression analysis is given in Table S1. The full list of UP- and DOWN-regulated DEG-associated pathways (Enrichment analysis) is presented in Supplementary Tables S2 and S3.

## 3. Discussion

In this study, we for the first time detected a significant acceleration in myoblast differentiation and concomitant decrease in the proliferation rate of myoblasts isolated from rat soleus muscle after mechanical unloading (HS). We analyzed the level of proliferation in soleus-derived myoblasts after both short-term and long-term HS (Figure 2a). There was a trend towards a reduction in the proliferation rate of myoblasts isolated from rat soleus muscle after 1-day HS (Figure 2c) and a significant decrease in myoblast proliferation after 7 days of HS (Figure 2c). Similar results were obtained by Mitchell and co-authors (2004) who measured the level of proliferation of myoblasts derived from murine skeletal muscles after 14-day hindlimb unloading [20]. There was also a decrease in soleus-derived myoblast proliferation (as assessed by BrdU staining) after 10-day hindlimb unloading [27]. In addition, Mitchell and co-authors (2004) showed that differentiation of cultured myoblasts isolated from atrophied muscles after 14 days of HS is significantly slowed down relative to myoblasts isolated from control muscles [20]. However, on the contrary, we found an accelerated differentiation of soleus-derived myoblasts after mechanical unloading (Figure 2d). In our experiment, starting from the 1st day of HS, mRNA expression of the key markers of myoblast fusion, *Mymk* and *Mymx*, was significantly increased in the cultured myoblasts (Figure 3i,j). In addition, the expression of various myosin heavy chain (MyHC) isoforms was observed in soleus-derived myoblasts cultivated in a primary growth medium after 7 days of HS (Figure 3e–h). Moreover, immunocytochemical analysis of these myoblasts revealed the presence of slow isoform of MyHC and F-actin (Figure 3a). This indicates that long-term unloading promotes the earlier development of differentiation of myoblasts derived from rat soleus muscle. It is interesting to note that the expression of various isoforms of MyHC is usually observed at later stages of development of cultured myoblasts [28].

In addition, we analyzed the expression of MRFs and *Pax7* in myoblasts isolated from rat soleus muscle after mechanical unloading (Figure 3b–d). In control myoblasts we observed a concomitant expression of both PAX7 and MYOD (Figure 2b,d) which can be explained by an early time-point of myoblast culturing (24 h after purification). These data are in good agreement with a previous report showing that cells express both Pax7 and MyoD in day 4 cultures [29]. The expression levels of *Pax7* in myoblasts after 1 and 7 days of HS (Figure 3b) did not differ from that of the control group, however, PAX7^+^ cell population was significantly reduced after 7 days of HS (Figure 2b,d). We found an increase in the expression of *Myf5*, *Mrf4*, *MyoD,* and *MyoG* in the soleus-derived myoblasts after 1 day of HS and more pronounced increase in the mRNA expression of these MRFs following 7-day HS (Figure 3c,d and Figure S1). The proportion of MYOD^+^ cells in the soleus-derived myoblasts was significantly reduced after 1-day HS but significantly increased after 7-day unloading compared to the control values (Figure 2b,d). It was previously shown that two-week HS of mice can induce a decrease in *Myf5* expression and number of MYOD^+^ cells but does not affect *Pax7* mRNA expression in cultured myoblasts derived from hindlimb skeletal muscles [20]. In addition, Guitar et al. (2018) have demonstrated that 7-day limb immobilization in mice leads to an increase in the expression of *Pax7* and *MyoD* in skeletal muscle SCs and concomitant reduction in the total number of SCs [24]. In the present study, a decrease in the proportion of PAX7^+^ cells and an increase in the proportion of MYOD^+^ cells (Figure 2d) following mechanical unloading (7 days) indicates an earlier myogenic differentiation of the soleus-derived myoblasts compared to control conditions or exposure to shorter periods of unloading (24 h). Some discrepancies between the data obtained in the present study and previously published reports may be due to the fact that in the present work myoblasts were isolated exclusively from soleus muscle, while in other studies (Guitar et al. (2018) and Mitchell et al. (2004)) [20,24] primary myoblasts were isolated from a pool of different skeletal muscles (gastrocnemius, soleus, tibialis anterior, extensor digitorum longus and quadriceps femoris). It is known that the number and composition of SCs in various types of skeletal muscles can significantly differ [30]. Moreover, a response of different hindlimb muscles to mechanical unloading also varies with soleus muscle being more susceptible to the effects of unloading conditions [31].

Thus, we found that a relatively long period of mechanical unloading (7 days) leads to the enhanced myogenic differentiation of myoblasts derived from rat soleus muscle, while the proliferation rate of these cells is reduced.

Using whole-transcriptome analysis, we evaluated the effect of short-term and long-term exposure of rats to simulated microgravity (HS) on the expression of the key components of various signaling pathways in myoblasts isolated from soleus muscle. After the first day of HS, the expression of the key components of the myogenesis pathway in soleus-derived myoblasts was suppressed, however after 7 days of HS the expression of gene clusters involved in myogenesis was activated (Figure 4b). Interestingly, after short-term mechanical unloading some discrepancies in soleus-derived myoblasts were observed: the expression of the key components of the myogenesis pathway was suppressed, while mRNA expression of MRFs was increased (Figure 3 and Figure S1). However, after examining in detail the expression of which genes are downregulated under short-term HS, we found that the expression of gene clusters for BMP-signaling, ERK1/2-signaling, and pathways involved in response to TGF-β is suppressed (Table 1). According to literature data, suppression of the activity of these signaling pathways leads to premature myogenic differentiation [32,33]. At the early stages of SCs activation, BMP signaling pathway promotes an increase of the pool of proliferating cells and prevents premature differentiation in myoblasts [33]. We found a significant suppression of the expression of genes of BPM signaling pathway in soleus-derived myoblasts after both 1 and 7 days of HS (Table 1 and Table 3).

It is also known that a decrease in the activity of the ERK1/2 signaling pathway leads to a reduction in the proliferation rate of myoblasts [34]. In addition, it has been shown that inhibition of the ERK1/2 signaling pathway leads to the activation of calcium-dependent mechanisms causing robust differentiation of activated SCs [32]. In the present study, we observed a decrease in the expression of genes of the ERK1/2 pathway in myoblasts isolated from the soleus muscle after 1 day of HS (Table 1). TGF-β is known to inhibit the proliferation and differentiation of myoblasts [35]. A significant decrease in the expression of genes responsible for the cell response to TGF-β (Table 1) probably contributed to the premature myogenic differentiation after short-term mechanical unloading.

In addition, in soleus-derived myoblasts 1-day HS resulted in a significant suppression of the expression of genes that negatively regulate Wnt signaling pathway (Table 1). It was previously shown that stimulation of the canonical Wnt pathway at the early stage of regeneration leads to a premature differentiation of myoblasts and contributes to the depletion of the SC pool [36]. We can speculate that some activity of this signaling pathway also contributes to the premature differentiation of myoblasts. It has been also shown that overexpression of Wnt5a protein causes an increase in myoblast proliferation [37] and, according to our data, the expression of Wnt5a gene was suppressed in soleus-derived myoblasts after 1-day HS. Consequently, the suppression of Wnt5a gene expression could contribute to a decrease in myoblast proliferation, which was observed in our study. Further research will be needed to confirm this hypothesis.

Thus, we have revealed short-term effects of unloading on the suppression of genes of a number of signaling pathways in soleus-derived myoblasts. Apparently, the suppression of the expression of the above-mentioned genes can lead to an increase in the expression of MRFs and ultimately contribute to accelerated myoblast differentiation.

After 7-day HS, expression levels of genes regulating myoblast differentiation, transport of calcium ions, Notch-signaling, and apoptotic processes are upregulated in rat soleus-derived myoblasts (Table 2).

These data correlate well with the increases in the expression levels of MRFs, myoblast fusion markers, as well as embryonic and “mature” myosin isoforms (Figure 3) in myoblasts isolated from rat soleus muscle after HS and cultured in primary growth medium.

According to literature, activation of the Notch signaling pathway promotes the activation of myoblast proliferation [38,39]. We revealed an increase in the expression of some genes of the Notch signaling pathway against the background of the reduced proliferation rate of myoblasts derived from soleus muscle after mechanical unloading (Table 2). An increase in the expression of *notch-3* gene was found among the genes of this signaling pathway. Previously it was shown that Notch-3 enhances the processes of replenishing SCs pool (self-renewal) and can act as a repressor of Notch1 [40] and probably contributes to the suppression of proliferation of activated SCs.

The expression of genes controlling the Wnt and BMP signaling pathways in soleus-derived myoblasts was suppressed after 7 days of HS, as well as after 1-day HS (Table 1 and Table 3). This fact may indicate suppression of proliferative processes which is confirmed by a decrease in the number EdU^+^ cells observed in the present study. It is interesting to note that in soleus-derived myoblasts after 7 days of HS, an activation of the Notch signaling pathway was observed which could promote myoblasts proliferation [41,42] against the background of a significant decrease in the myoblast proliferation rate. In addition, the Wnt signaling pathway, which is known to regulate myoblast differentiation [36], was suppressed in soleus-derived myoblasts together with the apparent signs of cell differentiation. It is known that a switch from the Notch signaling pathway to the canonical Wnt pathway is necessary for normal development of myoblast differentiation [43]. As we observed in the soleus-derived myoblasts after 7-day HS such a switch does not occur, while the process of myoblast differentiation is already underway. These data suggest that mechanical unloading (HS) promotes enhanced differentiation of soleus-derived myoblasts. In addition to regulatory signaling pathways, we considered changes in the expression of gene clusters directly involved in the processes of differentiation and regeneration (Figure 5). We show here that there are two phases in a response of soleus-derived myoblasts to mechanical unloading. During the first phase that lasts up to the 7th day of HS we detect a significant increase in MYOD^+^ subpopulation of cultured myoblasts (Figure 2b,d) and upregulation of regulatory pathways that stimulate muscle stem cells myogenic differentiation (Figure 4 and Figure 5). It is interesting to note that in Duchenne muscular dystrophy (DMD) the repeated cycles of myofiber degeneration and regeneration occur until SCs pool is depleted [5,11,44,45]. In the present study, changes in myoblast activity in response to mechanical unloading were only transitory and by day 14 of HS all upregulated pro-myogenic pathways were downregulated back to the control/HS1 levels (Figure 4 and Figure 5). Interestingly, Chemello et al. (2020) [11], using a mouse model of DMD (ΔEx51), performed snRNA-seq (single-nucleus RNA sequencing) on nuclei isolated from tibialis anterior muscle of wild-type (WT) mice and DMD (ΔEx51) mice and compared cell populations in dystrophic and WT skeletal muscles. They identified three clusters of nuclei related to skeletal muscle regeneration, and a cluster of cells positive for embryonic and perinatal myosin isoforms, *Myh3*, *Myh8*, and fusogenic factor *Mymk* was shown exclusively in DMD (ΔEx51) dystrophic fibers. Our data fit these observations: during the first 7 days of HS we have detected the upregulation of pathways related to muscle regeneration and fusogenic genes expression (Figure 3d,i,j) in atrophying soleus muscle (Figure 4 and Figure 5), but unlike DMD dystrophic fiber, the upregulation in unloaded fiber was not continuous (Figure 4b and Figure S1) which may indicate muscle adaptation to the new physiological situation in order to prevent the pathological acceleration of unloading-induced stem cell niche wasting.

It is also important to note that accumulation of calcium ions is observed in muscle fibers under myodystrophy conditions [46]. In myoblasts isolated from dystrophic muscle, faster differentiation accompanied by an early decrease in *MyoD* expression along with increased *MyoG* expression is observed [46,47]. In the present study, transcriptome analysis showed a significant change in the expression of genes of signaling pathways responsible for the regulation of intracellular calcium concentration and calcium-dependent signaling in soleus-derived myoblasts following short-term and long-term hindlimb unloading (Table 1, Table 2 and Table 3). This data may indirectly indicate that unloading induced changes in calcium concentration in soleus-derived myoblasts. It can be assumed that, just as in the case of soleus muscle fibers [48], the accumulation of calcium ions occurs in SCs under unloading conditions. It was previously shown that the activation of calcium-dependent signaling pathways can lead to rapid differentiation of activated myoblasts [32]. It can be suggested that under unloading conditions the accumulation of calcium ions in soleus-derived myoblasts may result in significant changes in the regulation of both cell proliferation and cell differentiation.

## 4. Materials and Methods

### 4.1. Experimental Design

**Figure 7:**
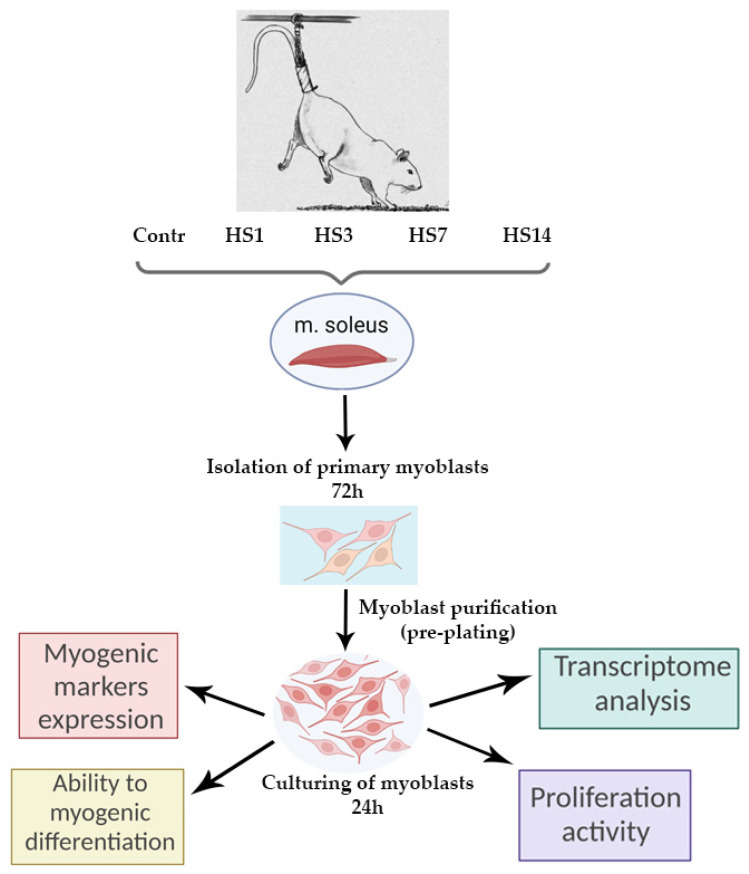
The experimental design.

Mechanical unloading was performed using a well-validated Morey–Holton hindlimb suspension (HS) model as previously described [25]. Thirty-two 3-month-old male Wistar rats (180 ± 5 g) were obtained from the certified Nursery for laboratory animals of the Institute of Bioorganic Chemistry of the Russian Academy of Sciences (Pushchino, Moscow region). The rats were housed in a temperature-controlled room on a 12:12-h light-dark cycle with food pellets and water provided ad libitum. The animals were randomly assigned to the following 4 groups (*n* = 8/group): (1) vivarium cage control (Contr), (2) 1-day hindlimb suspension (HS1), (3) 3-day hindlimb suspension (HS3), (4) 7-day hindlimb suspension (HS7), and (5) 14-day hindlimb suspension (HS14) (Figure 7). Under isoflurane anesthesia, soleus muscles from control and unloaded rats were surgically excised from both hindlimbs using standardized dissection methods, weighed on an analytical balance and then used for isolation of the pool of muscle SCs. After muscle excision, the rats were euthanized by decapitation under deep isoflurane anesthesia (4%).

### 4.2. Isolation of SCs

Soleus muscles were subjected to both mechanical and enzymatic dissociation according to the protocols described previously [49] with minor changes. Isolated muscles were placed into an enzymatic solution, mechanically disrupted with scissors, and digested for 60 min at 37 °C in 5 mL filtered 0.1% collagenase-1 (cat. C0130, Sigma-Aldrich, Saint Louis, MO, USA). Then, the obtained suspension was centrifuged for 5 min at 1000× *g* to remove collagenase and cell debris after digestion. The obtained supernatant was then discarded. The pellet was resuspended using sterile pipette tips in 2.5 mL of washing medium (DMEM supplemented with 10% horse serum (cat. 16050122, Gibco, ThermoFisher Scientific, Waltham, MA, USA). The suspension was pipetted for several minutes to isolate SCs and then centrifuged at 300× *g* for 5 min. The supernatant was transferred to a fresh tube. This stage was performed twice. The double-collected supernatant was centrifuged for 10 min at 1000× *g* to discard debris. After that, the resultant supernatant was discarded, and the pellet was placed in a growth medium (DMEM supplemented with 1% penicillin-streptomycin, 1% L-glutamine, 1% chicken fetal serum (cat. 092850145, MP Biomedicals, Santa Ana, CA, USA), 20% FBS (cat. 26140079, Gibco, ThermoFisher Scientific, Waltham, MA, USA), 10% horse-serum (cat. 16050122, Gibco, ThermoFisher Scientific, Waltham, MA, USA) on cell culture dishes covered with Geltrex (1:100 in cold DMEM, 1.5 h at 37 °C, cat. A1413202, Gibco, ThermoFisher Scientific, Waltham, MA, USA). Forty-eight h after the initial tissue digestion the cells became visible under a microscope, and half of a growth medium was changed. Seventy-two hours after the initial tissue digestion the adhesion selection method (pre-plating) [49,50] was used to separate the population of satellite cells from fibro-adipogenic progenitors (FAPs) (Figure 7). In brief, the cells were detached with trypsin using a standard method and centrifuged (300× *g*, 5 min). The pellet was resuspended in a medium containing 4.5 g/L D-glucose, 20% FBS, 1% penicillin-streptomycin, and 1% L-glutamine. The solution was transferred to a culture dish without Geltrex and placed to the CO_2_ incubator for 30–40 min. FAPs got attached to the dish, while SCs remained in solution. After pre-plating floating SCs were harvested, centrifuged (300× *g*, 5 min), mixed with growth medium and seeded in Geltrex-coated plates. The cells were cultured for 24 h under a humidified atmosphere with 5% CO_2_ at 37 and then used for RT-PCR, RNA-Seq, and immunocytochemical analysis (Figure 7). To check myoblast’s ability to form myotubes, myogenic differentiation was induced using a standard protocol. Proliferation medium was replaced with differentiation medium (DMEM medium supplemented with 4.5 g/L D-glucose, L-glutamine, penicillin-streptomycin, 2% of horse serum).

### 4.3. Proliferation Assay

Myoblast proliferation was assessed using the Click-IT EdU Alexa Fluor 555 Imaging Kit (cat. C10338, Thermo Fisher Scientific, Waltham, MA, USA). Briefly, cells were incubated with 3 µM EdU overnight to label newly synthesized DNA. The cells were fixed and permeabilized before Alexa Fluor 555 conjugation of the incorporated EdU. Microscopy images were obtained using an Olympus IX83P2ZF3 inverted fluorescent microscope with a DP74 camera (Olympus, Tokyo, Japan) and 20× magnification. Image analysis was carried out using the Cell Sens Imaging Software (Olympus, Tokyo, Japan).

### 4.4. RNA Isolation, cDNA Synthesis, and qRT-PCR

Non-differentiated SCs (0 day) were lysed using ExtractRNA kit (cat. BC032, Evrogen, Moscow, Russia). RNA concentration was measured using Nanodrop 3300 (Thermo Fisher Scientific, Waltham, MA, USA). Reverse transcription was performed from 500 ng RNA using Moloney Murine Leukemia Virus Reverse Transcriptase (MMLV RT) kit (cat. SK021, Evrogen, Moscow, Russia) according to manufacturer’s instructions. Real-time PCR was performed using qPCRmix-HS SYBR+LowROX protocol (cat. PK156, Evrogen, Moscow, Russia). Primers for real-time PCR were designed via NSBI BLAST program. PCR primers used for RNA analysis are shown in Table 4. Real-time PCR data are presented as arbitrary units of mRNA expression normalized to house keeping GAPDH expression and to expression levels in the reference sample using ∆∆Ct method.

### 4.5. Immunocytochemistry

Non-differentiated SCs were plated on coverslips. The cells were fixed in 4% paraformaldehyde for 10 min and then permeabilized with 0.02% Triton X-100 for 5 min. Cells were then blocked with 5% Bovine Serum Albumin (cat. A9647, Sigma-Aldrich, Saint Louis, MO, USA) for 30 min and incubated with primary antibodies against MYOD (cat. 554130, BD Bioscience, San Jose, CA, USA), PAX7 (Developmental Studies Hybridoma Bank, Iowa City, IA, USA), MHC slow (cat. M8421, Sigma; Sigma-Aldrich, Saint Louis, MO, USA), Phalloidin-iFluor 488 Reagent (cat. ab176753, Abcam, Cambridge, UK) and secondary Alexa Fluor 488-coupled goat-anti-mouse antibodies (cat. A28175, ThermoFisher Scientific, Waltham, MA, USA). Nuclei were counterstained with 4′,6-diamidino-2-phenylindole (DAPI) (cat. D1306, Molecular Probes, Waltham, MA, USA). Microscopy images were obtained using Zeiss Axio Observer Z1 (CarlZeiss, Oberkochen, Germany).

### 4.6. RNA-seq Analysis

RNA sequencing analysis was performed on cultured myoblasts (24 h after purification/pre-plating) derived from rat soleus muscle of control and hindlimb-unloaded rats (1, 3, 7 and 14 days of unloading). Libraries for RNA sequencing were prepared using TruSeq Stranded mRNA kit (cat. RS-122-2101, Illumina Inc., San Diego, CA, USA) according to manufacturer’s manual. Libraries were quantified with 4150 TapeStation system (Agilent, Santa Clara, CA, USA) using High Sensitivity DNA ScreenTape Analysis. Sequencing was performed on Illumina NextSeq 2000 (100 cycles). The quality of raw RNA-seq data was evaluated using FastQC tool (v0.11.9). Then reads were mapped on rat genome using aligner STAR (v2.604a) with reference genome mRatBN7.2. Mapped reads were count with the featureCounts program (v1.6.4) [51]. Alignment quality control was done with MultiQC tool (v1.10.1). Differentially expressed genes (DEGs) were determined using R package DESeq2. Genes *p*-values were adjusted using the Benjamini–Hochberg procedure and filtered with *p* < 0.01, only genes with log2 fold change > 1.5 were considered as differentially expressed. Then we performed fast gene set enrichment analysis (FGSEA) and GO Enrichment Analysis of gene sets. Cluster analysis of the gene set was done using the Phantasus (v1.11.0) online tool using the k-mean method. Then we annotated the resulting clusters with Enrichr online tool [52]. We used KEGG and MSigDB Hallmark databases to determine signaling pathways.

The sequence data presented in the study are openly available in GEO database. Available online: https://www.ncbi.nlm.nih.gov/geo/query/acc.cgi?acc=GSE201281 (accessed on 22 April 2022).

### 4.7. Statistical Analysis

Statistical analysis was performed using GraphPad Prism 8 software (GraphPad Software, San Diego, CA, USA, www.graphpad.com (accessed on 1 March 2022)). qRT-PCR and proliferation assay data are presented as mean ± SEM. One-way analysis of variance (ANOVA) was used to assess potential differences between the groups. Tukey post-hoc analysis was used to adjust for multiple comparisons. A level of significance of *p* < 0.05 (* *p* < 0.05, ** *p* < 0.01, *** *p* < 0.001, **** *p* < 0.0001) was established in the study.

## 5. Conclusions

We found that short- and long-term mechanical unloading (HS) can exert different effects on myoblasts derived from rat soleus muscle. According to the whole-transcriptome analysis, a decrease in the expression of a number of genes of signaling pathways responsible for myogenesis is revealed in myoblasts with short-term mechanical unloading, while longer exposure to unloading on the contrary, leads to an increase in the expression of the key genes of these signaling pathways. In this case soleus-derived myoblast proliferation is suppressed. In addition, increased expression of MRFs and myoblast fusion markers under mechanical unloading was revealed, and the expression of myosin genes was observed (with a longer period of unloading), indicative of myoblast differentiation. No such processes were detected in myoblasts derived from soleus muscle of the control rats. We have also shown that there are two stages of myoblast response to hindlimb unloading. During the first stage, which lasts up to day 7 of unloading, the regeneration program is activated; however, by day 14 of unloading this response is inactivated and the status of soleus-derived myoblasts returns to the control levels. These results provide an important background for further investigation of molecular and cellular targets for prevention of skeletal muscle wasting during long-term mechanical unloading.

## Figures and Tables

**Table 1:** Pathways associated with the DEGs downregulated in HS1 vs. CONTR (*p* < 0.01).

Pathways	Number of DEGs	Genes
Extracellular matrix organization GO: 0030198	34	*Col1a1*, *Tgfbi*, *Pdgfra*, *Col6a1*, *Cyp1b1*, *Has2*, *Grem1*, *Adamtsl3*, *Col16a1*, *Loxl3*, *Smoc2*, *Col8a1*, *Eng*, *Itga8*, *Adamts9*, *Col14a1*, *Olfml2a*, *Adamts2*, *Lum*, *Adamts8*, *Kazald1*, *Fbln5*, *Itgb3*, *Acan*, *Has1*, *Csgalnact1*, *Fmod*, *Dpt*, *Foxc2*, *Mmp3*, *Adamts16*, *Ptx3*, *Il6*, *Foxc1*
Striated muscle tissue development GO: 0014706	42	*Pdgfra*, *Grem1*, *Dcn*, *Ifitm3*, *Wnt5a*, *Eng*, *Adamts9*, *Col14a1*, *S100b*, *Meox2*, *Aldh1a2*, *Nr2f2*, *Erbb3*, *Pdgfrb*, *Shox2*, *Sox8*, *Dll1*, *Foxc2*, *Igf1*, *Eya2*, *Myh15*, *Csrp3*, *Foxc1*
Regulation of cytosolic calcium ion concentration GO: 0051480	22	*Pdgfra*, *Thy1*, *Bdkrb2*, *Wnt5a*, *Ramp3*, *Ptgir*, *Synpo*, *Htr2a*, *Itgb3*, *Adcy5*, *Ednrb*, *Cacna1c*, *Cmklr1*, *Avp*, *Lpar3*, *Pth1r*, *Ackr4*, *Ptgfr*, *Cacna1g*, *Ednra*, *Kdr*, *C5ar2*
Regulation of cellular response to growth factor stimulus GO: 0090287	20	*Fbn1*, *Vegfd*, *Grem1*, *Dcn*, *Smoc2*, *Acvrl1*, *Xdh*, *Wnt5a*, *Eng*, *Itga8*, *Fgf10*, *Smad6*, *Fbn2*, *Dll1*, *Hjv*, *Msx1*, *Vsir*, *Kdr*, *Tmem204*, *Cdkn1c*
Transmembrane receptor protein serine/threonine kinase signaling pathway GO: 0007178	20	*Fbn1*, *Grem1*, *Acvrl1*, *Wnt5a*, *Lrrc32*, *Eng*, *Itga8*, *Ror2*, *Fgf10*, *Cilp*, *Smad6*, *Fbn2*, *Tmem100*, *Hjv*, *Msx1*, *Vsir*, *Rbpms*, *Kdr*, *Lgals9*, *Cdkn1c*
Muscle cell differentiation GO:0042692	18	*Pdgfra*, *Grem1*, *Ifitm3*, *Enpp3*, *Nfatc4*, *Eng*, *Itga8*, *Col14a1*, *Fgf10*, *Ednrb*, *Pdgfrb*, *Shox2*, *Dll1*, *Igf1*, *Msx1*, *Casp1*, *Tmem204*, *Csrp3*
ERK1 and ERK2 cascade GO: 0070371	15	*Pdgfra*, *Ramp3*, *Ece1*, *Fgf10*, *Htr2a*, *Itgb3*, *Pdgfrb*, *Avp*, *Igf1*, *Ednra*, *Kdr*, *Lgals9*, *Il6*, *Hand2*, *C5ar2*
Cytokine-mediated signaling pathway GO: 0019221	14	*Ifitm3*, *Il1rn*, *Wnt5a*, *Il11ra1*, *Il33*, *Ifitm1*, *Crlf1*, *Cxcl6*, *Ackr4*, *Slit3*, *Il20rb*, *Casp1*, *Il6*, *Foxc1*
Response to transforming growth factor β GO: 0071559	13	*Fbn1*, *Col1a1*, *Penk*, *Acvrl1*, *Wnt5a*, *Lrrc32*, *Eng*, *Itga8*, *Cilp*, *Smad6*, *Fbn2*, *Star*, *Cdkn1c*
BMP signaling pathway GO: 0030509	12	*Fbn1*, *Grem1*, *Acvrl1*, *Wnt5a*, *Eng*, *Ror2*, *Smad6*, *Tmem100*, *Hjv*, *Msx1*, *Vsir*, *Kdr*
Regulation of Wnt signaling pathway GO: 0030111	12	*Col1a1*, *Grem1*, *Wnt5a*, *Nfatc4*, *Plpp3*, *Ror2*, *Tnn*, *Fgf10*, *Nkd2*, *Cthrc1*, *Dkk2*, *Gsc*
Tumor necrosis factor production GO: 0032640	11	*Wnt5a*, *Nfatc4*, *Twist2*, *Il33*, *Spon2*, *Ptafr*, *Igf1*, *Vsir*, *Lgals9*, *Il6*, *C5ar2*
Calcium-mediated signaling GO: 0019722	10	*Nfatc4*, *Erbb3*, *Ednrb*, *Cacna1c*, *Cmklr1*, *Tmem100*, *Ackr4*, *Ptgfr*, *Igf1*, *Kdr*
Cellular response to mechanical stimulus GO: 0071260	8	*Col1a1*, *Aqp1*, *Eng*, *Il33*, *Itgb3*, *Piezo2*, *Ednra*, *Casp1*
Phosphatidylinositol 3-kinase signaling GO: 0014065	8	*Dcn*, *Ror2*, *Hgf*, *Htr2a*, *Erbb3*, *Pdgfrb*, *Igf1*, *Kdr*
Apoptotic process involved in development GO: 1902742	5	*Slit3*, *Foxc2*, *Robo2*, *Hand2*, *Foxc1*

**Table 2:** Pathways associated with the DEGs upregulated in HS7 vs. CONTR (*p* < 0.01).

Pathways	Number of DEGs	Genes
Striated muscle tissue development GO: 0014706	42	*Tgfb2*, *Heyl*, *Sox11*, *Erbb3*, *Plagl1*, *Myo18b*, *Myog*, *Srpk3*, *Msc*, *Myom1*, *Tnnt2*, *Dmd*, *Actc1*, *Acta1*, *Tnnc1*, *Rgs2*, *Fhod3*, *Notch1*, *Gja5*, *Dll1*, *Stac3*, *Myh7*, *Cacna1s*, *Actn2*, *Tnni1*, *Mylpf*, *Lmod3*, *Chrnd*, *Tenm4*, *Myh15*, *Neb*, *Myom2*, *Smyd1*, *Wt1*, *Hey2*, *Myoz2*, *Nrap*, *Sox6*, *Col19a1*, *Csrp3*, *Mylk3*, *Rxrg*
Muscle cell differentiation GO: 0042692	37	*Itga8*, *Tmod1*, *Myo18b*, *Myog*, *Mymx*, *Tnnt2*, *Dmd*, *Actc1*, *Acta1*, *Olfm2*, *Ldb3*, *Pdgfb*, *Rgs2*, *Mymk*, *Fhod3*, *Notch1*, *Dll1*, *Stac3*, *Cacna1s*, *Actn2*, *Trim72*, *Casq2*, *Tnnt1*, *Tnnt3*, *Lmod3*, *Cfh*, *Neb*, *Mypn*, *Smyd1*, *Ankrd23*, *Wt1*, *Hey2*, *Myoz2*, *Nrap*, *Sox6*, *Csrp3*, *Mylk3*
Actomyosin structure organization GO: 0031032	19	*Tmod1*, *Tnnt2*, *Actc1*, *Acta1*, *Ldb3*, *Wnt11*, *Fhod3*, *Actn2*, *Casq2*, *Tnnt1*, *Tnnt3*, *Lmod3*, *Neb*, *Mypn*, *Ankrd23*, *Myoz2*, *Nrap*, *Csrp3*, *Mylk3*
Calcium ion transport GO: 0006816	19	*Tgfb2*, *Vdr*, *Cemip*, *Cacng6*, *Dmd*, *Srl*, *Pdgfb*, *Atp1a2*, *Stac3*, *Ryr3*, *Cacna1s*, *Casq2*, *Slc24a3*, *Hrc*, *Cacnb1*, *Camk2b*, *Nos1*, *Xcl1*, *Dhrs7c*
Myotube differentiation GO: 0014902	12	*Myog*, *Mymx*, *Dmd*, *Acta1*, *Mymk*, *Notch1*, *Stac3*, *Cacna1s*, *Trim72*, *Lmod3*, *Smyd1*, *Csrp3*
Notch signaling pathway GO: 0007219	11	*Tgfb2*, *Heyl*, *Notch3*, *Postn*, *Tp63*, *Notch1*, *Dll1*, *Dtx4*, *Hey2*, *Tspan15*, *Grip2*
Potassium ion transport GO:0006813	11	*Fxyd1*, *Atp1a2*, *Adora1*, *Gja5*, *Actn2*, *Casq2*, *Slc24a3*, *Kcnma1*, *Nos1*, *Scn4a*, *Kcna7*
Cellular response to transforming growth factor β stimulus GO: 0071560	11	*Tgfb2*, *Sox11*, *Itga8*, *Nrep*, *Itgb6*, *Postn*, *Adamtsl2*, *Cdkn1c*, *Cdh5*, *Sox6*, *Xcl1*
Regulation of apoptotic process involved in development GO: 1904748	4	*Tgfb2*, *Vdr*, *Notch1*, *Wt1*

**Table 3:** Pathways associated with the DEGs downregulated in HS7 vs CONTR (*p* < 0.01).

Pathways	Number of DEGs	Genes
Wnt signaling pathway GO: 0016055	15	*Grem1*, *Itga3*, *Cdh3*, *Dkk3*, *Fgf10*, *Hmga2*, *Bmp2*, *Klf4*, *Sulf2*, *Plpp3*, *Ror2*, *Ctnnd2*, *Nog*, *Dkk2*, *Ptpro*
Transmembrane receptor protein serine/ threonine kinase signaling pathway GO: 0007178	14	*Grem1*, *Itga3*, *Grem2*, *Fgf10*, *Bmp2*, *Myocd*, *Jade2*, *Ror2*, *Pparg*, *Msx2*, *Lgals9*, *Nog*, *Tmem100*, *Gdf10*
Canonical Wnt signaling pathway GO: 0060070	13	*Grem1*, *Cdh3*, *Dkk3*, *Fgf10*, *Bmp2*, *Klf4*, *Sulf2*, *Plpp3*, *Ror2*, *Ctnnd2*, *Nog*, *Dkk2*, *Ptpro*
Cytokine-mediated signaling pathway GO: 0019221	12	*Grem2*, *Il1rn*, *Il11ra1*, *Il1rl2*, *Cxcl3*, *F3*, *Cxcl6*, *Pparg*, *Ereg*, *Arg1*, *Il20rb*, *Foxc1*
Stem cell differentiation GO: 0048863	9	*Grem1*, *Hmga2*, *Myocd*, *Ednrb*, *Osr1*, *Msx2*, *Sema3e*, *Hand2*, *Foxc1*
BMP signaling pathway GO: 0030509	9	*Grem1*, *Itga3*, *Grem2*, *Bmp2*, *Ror2*, *Pparg*, *Msx2*, *Nog*, *Tmem100*

**Table 4:** Primers used for the qRT-PCR analysis.

Gene Name	Sequence (5′->3′)	GenBank
*Pax7*	5′-tataagagggagaaccccgga-3′ 5′-gctaatcgaactcactgaggg-3′	NM_001191984.1
*Myf5*	5′-ccctgatgtatcaaacgcatgt-3′ 5′-cgtgatccgatccactatgct-3′	NM_001106783.1
*Myf6*	5′-cttgagggtgcggatttcct-3′ 5′-cgcttgctcctccttcctta-3′	NM_013172.2
*MyoG*	5′-ggtcccaacccaggagatca-3′ 5′-acatatcctccaccgtgatgc-3′	NM_017115.3
*MyoD*	5′-tgctctgatggcatgatgga-3′ 5′-ctggacgcctcactgtagta-3′	NM_176079.2
*Gapdh*	5′-cggtgtgaacggatttggc-3′ 5′-ttgaggtcaatgaaggggtcg-3′	NM_017008.4
*Mymx*	5′-gatcgctgctatcacgcct-3′ 5′-ctcacgtcttgggagctcag-3′	NM_001399466.1
*Mymk*	5′-tctttgtggcgttctcccat-3′ 5′-cagggctgtcccatagatgc-3′	NM_001399315.1

## Data Availability

The data presented in the study are available upon reasonable request from the corresponding author.

## Supplementary Materials

The following supporting information can be downloaded at: https://www.mdpi.com/article/10.3390/ijms23169150/s1.
