# Supplementary figures and images for: Cultured Myoblasts Derived from Rat Soleus Muscle Show Altered Regulation of Proliferation and Myogenesis during the Course of Mechanical Unloading
Source: Int J Mol Sci. 2022 Aug 15;23(16):9150. doi: 10.3390/ijms23169150 (PMC9409304; doi:10.3390/ijms23169150)

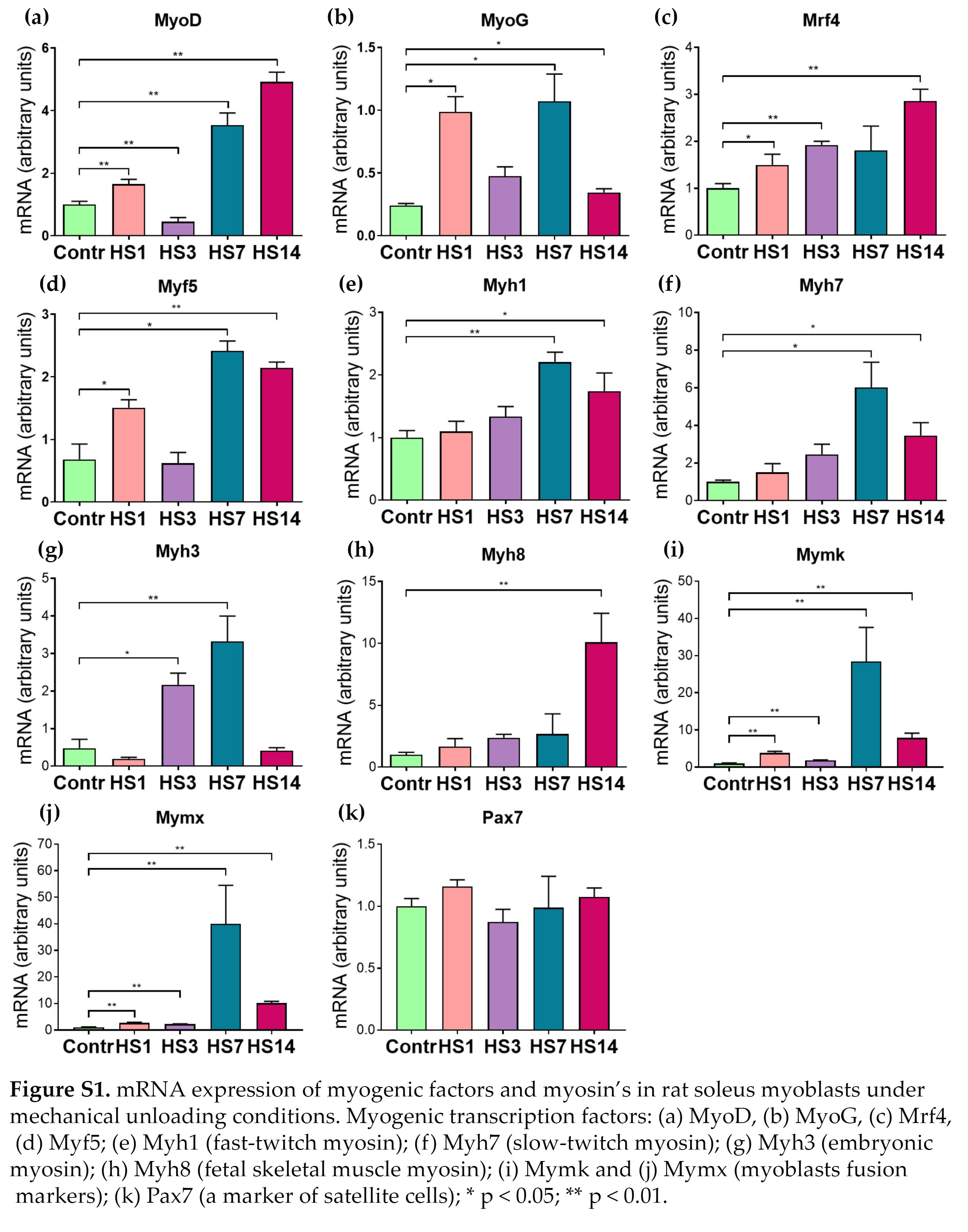

Supplement: Supplementary file 1 [file ijms-23-09150-s001.zip › ijms-1821942-supplementary/Figure S1.tif]

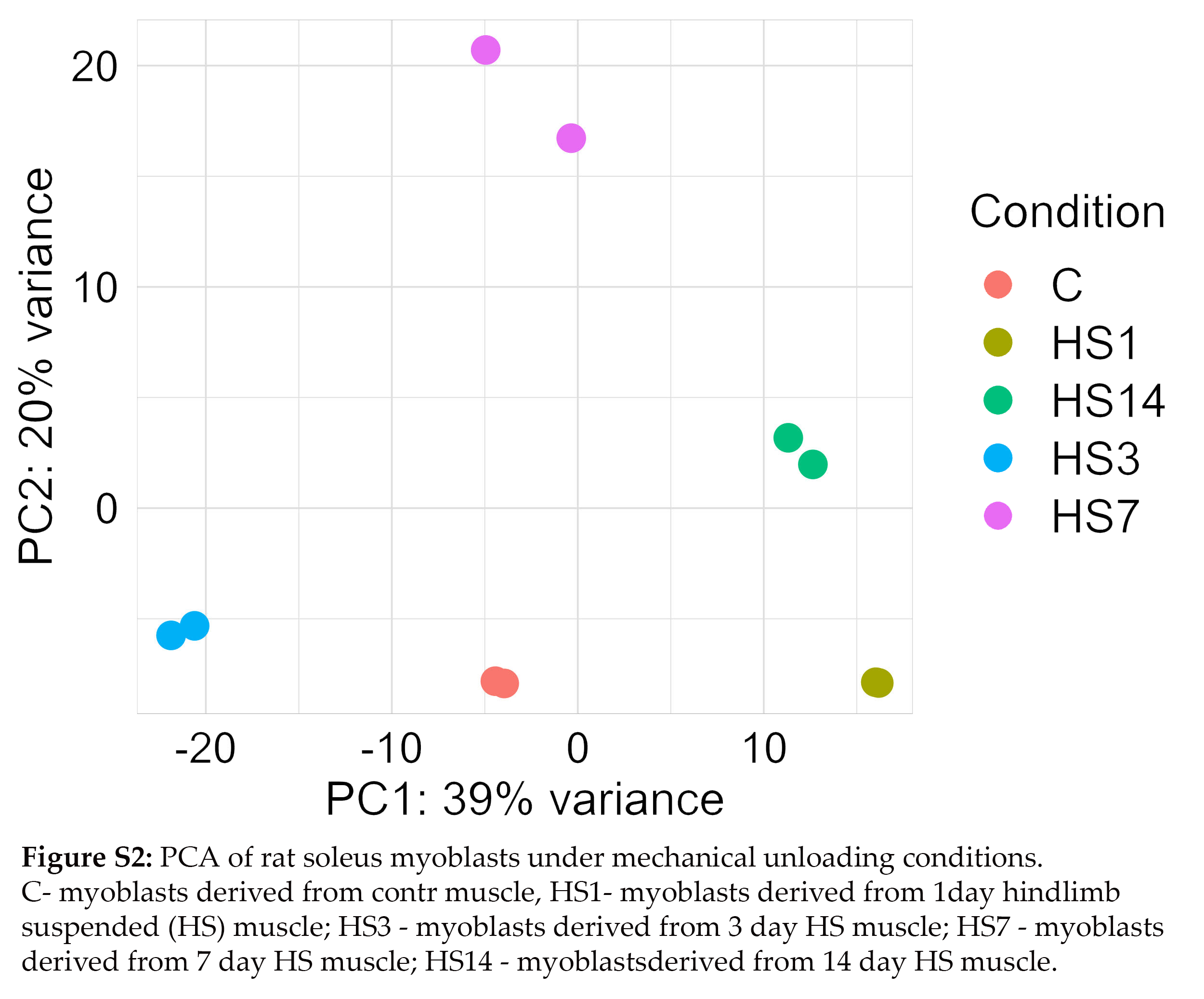

Supplement: Supplementary file 1 [file ijms-23-09150-s001.zip › ijms-1821942-supplementary/Figure S2.tif]
